# Genome Sequence of Abbey Lake Virus, a Novel Orthobunyavirus Isolated from China

**DOI:** 10.1128/genomeA.00433-14

**Published:** 2014-06-19

**Authors:** Ran Liu, Guilin Zhang, Yinhui Yang, Rongli Dang, Tongyan Zhao

**Affiliations:** a Center for Disease Control and Prevention of Xinjiang Military Command Region, Urumuqi, Xinjiang Uygur Autonomous Region, Zhong Guo, China; b Institute of Microbiology and Epidemiology, Beijing, Zhong Guo, China

## Abstract

The *Orthobunyavirus* genus of *Bunyaviridae* is a divergent group of medically important pathogens. Abbey Lake bunyavirus (Ab-BUNV) was newly isolated and identified in Xinjiang Province, northwestern China. The complete genome of Ab-BUNV was sequenced and is reported here, revealing that Ab-BUNV may represent a novel genotype in the genus *Orthobunyavirus*.

## GENOME ANNOUNCEMENT

The *Orthobunyavirus* genus of *Bunyaviridae* is a divergent group of zoonotic pathogens causing severe hemorrhagic fever disease and central nervous system infection, and it has attracted much medical concern. Orthobunyaviruses possess a negative-sense single-stranded tripartite RNA genome, consisting of large (L), medium (M), and small (S) segments, which encode an RNA-dependent RNA polymerase (RdRp), two envelope glycoproteins (Gn/Gc), and a nucleocapsid protein (NP), respectively. Orthobunyaviruses are widely distributed throughout large parts of Africa, Europe, and Asia. However, the circulation of orthobunyaviruses remains unclarified, especially for China (1); for now, Batai virus and Tahyna virus are the only two characterized viral isolates in mainland China (2, 3).

Abbey Lake bunyavirus (Ab-BUNV), originally isolated from *Culex* species in Xinjiang Uygur Autonomous Region, northwestern China, in 2013, was preliminarily identified as a member of the *Orthobunyavirus* genus of the *Bunyaviridae* family by reverse transcription amplification of a partial S segment (4). Interestingly, phylogenetic analysis indicated that Germiston virus, which was historically isolated in Africa, is the unique viral relative to Ab-BUNV (5).

For better identification of Ab-BUNV, a complete genome sequence covering all three segments was *de novo* generated using the Illumina HiSeq 2000 system. S and M segments (accession no. M19420.1 and M21951.1, respectively) of Germiston virus and an L segment (accession no. JX846606.1) of Batai virus strain MS50 were used as references for the sequence assembly (6). DNAStar SeqMan (version 7; Lasergene) and MEGA 5.10 were employed for the genomic sequence alignment. Overlapping primer sets were designed based on the sequences determined with the HiSeq 2000 to allow conﬁrmation by Sanger sequencing.

The full-length L segment of Ab-BUNV is 6,799 nucleotides, with a predicted RdRp of 2,238 amino acids, starting at nucleotide position 47 and including 36 nucleotides of the 3′ noncoding region. The entire M segment of the Ab-BUNV strain is 4,422 nucleotides, with a predicted glycoprotein precursor of 1,435 amino acids. The full-length S segment of the Ab-BUNV strain is 973 nucleotides, with a predicted NP of 233 amino acids.

Phylogenetic analysis showed that S, M, and L segments of Ab-BUNV are distinguished from reference Germiston virus or Batai virus MS50 segments, with low identities at the nucleotide (89%, 77%, and 73%) and amino acid(96%, 89%, and 80%) levels, respectively. Indeed, the data suggest that Ab-BUNV may represent a novel genotype within the *Orthobunyavirus* genus. The complete genome sequence reported here will help better understand the variation and evolution of Ab-BUNV and the other orthobunyaviruses.

### Nucleotide sequence accession numbers.

The tripartite genome sequence of Ab-BUNV has been deposited in GenBank under the following accession numbers: KJ710424 for the S segment, KJ710423 for the M segment, and KJ710425 for the L segment.
